# TLR-4 Inhibition Attenuates Inflammation, Thrombosis, and Stenosis in Arteriovenous Fistula in Yucatan Miniswine

**DOI:** 10.26502/fccm.92920280

**Published:** 2022-08-26

**Authors:** Vikrant Rai, Mohamed M Radwan, Sunil Nooti, Finosh G Thankam, Harbinder Singh, Devendra K. Agrawal

**Affiliations:** Department of Translational Research, Western University of Health Sciences, Pomona, CA 91766, USA

**Keywords:** Arteriovenous Fistula, AVF Failure, AVF Maturation, Chronic Inflammation, Early Thrombosis, Neointimal Hyperplasia, TLR-4 Inhibition, TAK-242

## Abstract

Arteriovenous fistula (AVF) is the preferred vascular access in hemodialysis patients; however, it is afflicted with a high failure rate. Chronic inflammation, excessive neointimal hyperplasia (NIH), vessel stenosis, early thrombosis, and failure of outward remodeling are the major causes of AVF maturation failure. Inflammatory mediator toll-like receptor (TLR)-4 plays a critical role in NIH, arterial thrombosis, and stenosis. We investigated the effect of TLR-4 inhibition on early thrombosis. Yucatan miniswine were used to create AVF involving femoral artery and femoral vein and treated with TLR-4 inhibitor TAK-242 with ethanol as the vehicle. The vessels were assessed after 12 weeks using histomorphometry, immunostaining, ultrasound, angiography, and optical coherence tomography. Inhibition of TLR-4 attenuated inflammation and early thrombosis in 50% of animals, and blood flow was present through AVF in 25% of animals. Thus, targeting TLR-4 to attenuate inflammation and early thrombosis might be a therapeutic approach to keep AVF patent and maintain blood flow through the outflow vein.

## 1. Introduction

Autologous arteriovenous fistula (AVF), a connection of an artery to a vein, is the preferred vascular access in end-stage renal disease patients who require hemodialysis because of its good long-term patency, evading the risk of central venous catheter-related complications, and a lower rate of complications compared to artificial graft [1–3]. The benefits of the AVF depend on the maturation of the fistula and it is afflicted with a high maturation failure rate [4]. Maturation of the AVF is characterized by outward vascular remodeling of the outflow vein facilitated by increased nitrous oxide release and elastin breakdown to facilitate vein enlargement. AVF maturation increases blood flow through the anastomosing artery and outflow vein with vein wall thickness and dilation to allow the use of the fistula for hemodialysis [1]. However, early thrombosis with poor blood flow results in AVF failure which might be either early or late failure [1, 4]. Maturation failure is characterized by narrowing or inadequate dilation and inadequate blood flow in the outflow vein which renders the fistula not useful for hemodialysis after initial adequate blood flow upon AVF creation. Excessive neointimal hyperplasia (NIH), early thrombosis, vessel stenosis, and failure of outward remodeling are the major causes of AVF non-maturation accounting for 60% of all newly created AVFs. 25–35% of the matured AVFs fail within two years and need treatment either by angioplasty or surgical intervention [5–8]. Early thrombosis is mainly due to juxta-anastomosis stenosis or the presence of an accessory vein (inflow problem) while outflow stenosis causes late thrombosis. Insufficient arterial inflow is another critical factor responsible for AVF non-maturation and failure [8]. Insufficient inflow in the artery is mainly caused due to obstructed lumen due to thrombosis, excessive NIH, plaque formation, and atherosclerosis [9]. Thus, inflow artery thrombosis and stenosis are critical in AVF failure.

Although the exact mechanisms of AVF maturation failure remain unclear, the role of inflammatory mediators and growth factors regulating NIH and vascular remodeling have been proposed [10, 11]. Initial inflammation and local hypoxia caused by surgical trauma to the artery and vein during fistula creation and then systemic inflammation play a crucial role in regulating AVF maturation and its failure. Inflammatory mediators including macrophages, infiltrating lymphocytes, macrophage migration inhibitory factor, chemokine (C-X-C motif) receptor (CXCR)-2, CXCR-4, interleukin (IL)-8, and monocyte chemotactic protein 1 (MCP-1), tumor necrosis factor (TNF)-α, IL-1β and growth factors such as vascular endothelial growth factor (VEGF)-A play a critical role in AVF failure [10]. These mediators also play a crucial role in regulating neointimal thickening, the proliferation of medial and intimal cells, and plaque formation [10, 12]. The venous NIH and failure of outward remodeling are attributed to inflammation, proliferation, migration, and phenotypic changes of vascular smooth muscle cells (VSMCs), and extracellular remodeling due to increased matrix metalloproteinases (MMPs) [13–16]. To improve clinical outcomes, pharmacological treatment with statins, anti-VEGF-A antibody, sirolimus, nitroglycerin, calcitriol, or perivascular elastase are potential strategies but so far the results are controversial and inconclusive [10, 17].

Triggering receptors expressed on myeloid cell (TREM)-1 and toll-like receptor (TLR)-4 play a critical role in NIH, vessel remodeling, cellular inflammation, migration, and proliferation of VSMCs, endothelial dysfunction, and atherosclerotic plaque formation and targeting TREM-1 and TLR-4 attenuate inflammation, plaque formation, thrombosis, and prevent restenosis [9, 18–23]. In this study, we investigated the effect of inhibiting TLR-4 by its pharmacological inhibitor TAK-242 [24] in attenuating inflammation, NIH, and stenosis in a swine model of AVF created between the femoral artery and femoral vein. TAK-242 is a selective inhibitor of TLR-4 signaling and attenuates its proinflammatory effects by selectively binding to TLR4 and interfering with the interactions between TLR4 and its adaptor molecules [25]. Thus inhibiting TLR-4 will attenuate the secretion of various proinflammatory cytokines involved in the pathogenesis of NIH, plaque formation, and stenosis [26]. We hypothesized that selective inhibition of TLR-4 with TAK-242 attenuates inflammation, and excessive NIH, regulates VSMCs migration and proliferation, thereby preventing early thrombosis and stenosis of the inflow femoral artery and this will increase blood flow through the outflow vein.

## 2. Materials and Methods

### 2.1 Animal Model

Four to seven months old Yucatan miniswine weighing between 20–30 Kilograms were purchased from Premier Bio-resources (CA, USA) for this study. All miniswine were kept at the animal facility at Western University of Health Sciences, Pomona, CA with 12 hours light and dark cycle at a temperature range of 72°-74° F. The swine were fed with the Mini-Pig Grower Diet (Test Diet # 5801) and allowed to drink water *ad libitum*. We used female pigs because they are less aggressive, safer, and easier to handle than males. Additionally, the commercially available male pigs are castrated for the safety of the personnel and castration induces hormonal changes which may be a confounding factor in the study. The miniswine with similar body weights were divided randomly into two groups: 30% Ethanol alone (control group, n=3) and 30% Ethanol + TAK242 (experimental group, n=4). TAK-242 was dissolved in 30% Ethanol. A total of 7 animals were used in this pilot study. Animal work was performed per the guidelines of the National Institutes of Health and USDA for the care and use of experimental animals. The animal research protocol No. R20IACUC038 of this study was approved by the Institutional Animal Care and Use Committee of the Western University of Health Sciences, Pomona, California.

### 2.2 Anesthesia and Recovery

The minipigs were first given a preanesthetic injection of Telazol (a combination of tiletamine and zolazepam) 2.5–5 mg/kg and xylazine 1–2 mg/kg subcutaneously. Following sedation of the pigs, they were moved to the operating room and intubated with an appropriately sized endotracheal tube. The anesthesia was maintained by inhalation of 1–3% isoflurane in oxygen and the pigs were mechanically ventilated. An indwelling intravenous catheter in the ear vein was placed and ringer lactate solution at a rate of 5mL/kg/hr was infused throughout the procedure. While the pigs were under anesthesia, we monitored and recorded every 15 minutes the level of sedation, percentage of isoflurane, oxygen flow rate, heart rate, respiratory rate, mucous membrane color, presence or absence of withdrawal reflex, and body temperature. During all survival procedures prophylactic antibiotics (cefazolin 1g IM) was given; buprenorphine were used for pain, and their preanesthetic sedation was reversed with flumazenil 0.01mg/kg. Once the surgery was completed, swine were monitored until they were standing steadily. Conscious level, recumbency, respiratory rate and character, and mucous membrane color were monitored as recovery parameters.

### 2.3 Creation of AVF in the Swine Model

The AVF was surgically created between the femoral artery (FA) and femoral vein (FV) in the minipigs. Briefly, the FA and FV were exposed and skeletonized, all the vein tributaries were ligated. The FA and FV were prepared for side-to-side anastomosis. A one cm incision was made on the medial side of the FA and the lateral side of the FV. The FA and FV were then anastomosed using a 6–0 proline. Just before completing the anastomosis 30% ethanol alone or TAK242 (3mg/kg) dissolved in 30% ethanol was injected into the FA and FV corresponding to the swine group. The anastomosis creation was then completed after waiting for 10 minutes from the time of injection. The FV distal to the AVF was ligated creating the functional end-to-side anastomosis. The AVF patency was assessed by visual inspection of the FV for dilation and arterial pulsations. Hemostasis was achieved and the inguinal incision was closed in a layered fashion using an absorbable vicryl suture of appropriate size, the skin was closed by a subcuticular 3–0 vicryl suture. The swine were given TAK-242 once daily at 0.1mg/kg (i.v.) for 6 days and then once weekly for 4 weeks as a maintenance dose.

### 2.4 Color Doppler Ultrasound

A preoperative color Doppler ultrasound (Phillips EPIQ −7 US system) was done to assess the diameter of the FA and FV, flow velocities, and flow volume in the FA. A postoperative ultrasound sonography (USG) of the FA and FV at the anastomosis site before sacrificing the minipig was done to assess the FA flow and diameter, outflow vein flow and diameter, and the flow velocities in the femoral vessels. Gain and imaging depth was adjusted per swine to obtain optimal USG images. Each site was evaluated for the peak systolic and end-diastolic velocity and blood flow at different locations in the artery and vein of the AVF, including the anastomosis site was evaluated.

### 2.5 Angiography

At the study endpoint, in an anesthetized pig, color Doppler ultrasound was followed by femoral angiography to assess the patency and blood flow in the fistula. The angiography was performed on the anastomosis and contralateral side (a reference image to compare with the AVF side) using a 7F Concierge guide catheter from Merit Medical USA through the carotid artery to access the FA of both sides. Briefly, angiography was performed by US-guided percutaneous needle puncture in the common carotid artery. The angiography catheter was advanced from the carotid artery into the descending aorta, and external iliac arteries to the FA of the AVF side as well as to the contralateral side. To visualize the AVF and contralateral FA patency, a contrast dye was then injected into the catheter while taking x-rays of the area of interest.

### 2.6 Optical Coherence Tomography (OCT)

OCT (OPTIS OCT machine from St. Jude Medical) was performed before euthanasia at the anastomosis site as well as at the contralateral FA via carotid approach. A 0.014-inch guidewire was positioned in the proximal FA. Subsequently, the OCT catheter (DragonflyTM DUO imaging catheter; Abbott USA) was advanced over the guidewire to the FA. Iopamidol contrast media was simultaneously injected during OCT pullback. The entire region of interest was scanned. The images were analyzed via Light Lab OCT imaging proprietary software (Light Lab Imaging/ Abbott). OCT was used to measure the inside diameter and the cross-sectional area of the vessels, to delineate the vessel’s wall anatomy (intima, media, and adventitia), the presence as well as characterization of NIH, thrombosis, neo-vascularization, luminal diameter, and percent diameter stenosis. The OCT of the AVF side was compared to the contralateral side FA. All radiological and other image analyses were done in a blinded manner.

### 2.7 Euthanasia, Tissue Harvest, And Processing

At the endpoint, euthanasia was performed by intravenous administration of a single dose of euthanasia solution (pentobarbital sodium (85 mg/kg) and phenytoin sodium (11 mg/kg)) while the pigs are under surgical anesthesia. Swine were observed for the absence of heartbeats and respiration for at least 10 min before tissue harvest. The groin was dissected to retrieve the AVF, FA proximal (PFA), and distal (DFA) to the anastomosis, FV proximal (PFV) to the site of anastomosis, tissues around the site of anastomosis, and contralateral FA and FV. The tissues were harvested for histomorphology studies in 10% formalin, for RT-PCR in RNA later, and for protein isolation at 4°C and stored at −80°C. The tissues were processed in a tissue processor through multiple changes of ethanol for dehydration and by multiple changes in xylene for clearing and embedded in paraffin wax to prepare tissue blocks. The paraffin wax blocks with tissues were sectioned at 5μm using a tungsten carbide knife (LeicaTM, Germany) in a Leica RM2265 rotary microtome (LeicaTM, Germany) and attached to slides. The tissue sections were heat blocked for 60 minutes and used for histology and immunostaining.

### 2.8 Hematoxylin and Eosin and Movat Pentachrome Staining

Hematoxylin and eosin (H&E) staining was done following the standard protocol in our lab. Briefly, after deparaffinization and rehydration through a series of xylene, alcohol, and distilled water, the tissue sections were stained with hematoxylin (45 seconds) followed by eosin (8–10 dips). The stained slides were mounted with xylene-based mounting media. Movat Pentachrome staining was done using a modified Russel Movat Pentachrome kit following the manufacturer’s protocol (Cat no. KTRMPPT from American MasterTech scientific laboratory supplies). Stained tissue sections were scanned at 100μm using a light microscope (Leica DM6). All scanned images were anonymously reviewed by two independent observers in a blinded manner.

### 2.9 Immunostaining

Immunohistochemistry (IHC) was performed using the peroxidase anti-peroxidase method using a secondary antibody conjugated to horseradish peroxidase (HRP). The paraffin fixed sections were deparaffinized, rehydrated, and antigen retrieved using 1% citrate buffer (Sigma Aldrich # C9999) before immunostaining as per the standard protocol in our laboratory. Briefly, the slides were washed with PBS after blocking and tissue was encircled using Pep Pen. The tissue samples were incubated with 3% hydrogen peroxide (Sigma Aldrich # H1009) for 15 minutes and washed with PBS for 5 minutes each three times. Blocking was done using the blocking solution from the Vectastain Elite ABC kit (Vector Labs) and the tissues were incubated for 1 hour at room temperature. After tipping off the blocking solution, the tissue sections were incubated overnight at 4°C with the primary antibodies (Supplementary Table 1). The slides were washed 3 times 5 minutes each with 1x PBS and then incubated with the secondary antibody from the corresponding Vectastain Elite ABC kit for 1 hour at room temp. The slides were rinsed 3 times with 1x PBS, followed by incubation with the Vectastain ABC horseradish peroxidase (HRP) for 30 minutes at room temp. The tissue sections were then rinsed with 1x PBS followed by incubation with 3,3′-diaminobenzidine (DAB) (Thermo Scientific, Cat # 34002) for 2 to 5 minutes until the development of brown color. Tissue sections were washed with water once and then stained with hematoxylin for 20–30 seconds. The slides were rinsed in running tap water for 5 minutes and mounted with a xylene-based mounting medium. The stained slides were imaged with a Leica DM6 microscope at a scale of 100 μm. The high magnification images from each tissue section were manually analyzed for the average stained area using Fiji Image J analyses [27]. Three sections for each swine were used for statistical analysis.

### 2.10 Quantitative Real-Time Polymerase Chain Reaction (qRT-PCR)

qRT-PCR was used to evaluate the effect of TAK-242 on TLR-4 downstream signaling by assessing the effect of TAK-242 on TRAF6, TIRAP, TRIF, TRAM, MyD88, and TLR-4 in-vitro and in-vivo. For in-vitro studies, VSMCs isolated from microswine arteries were treated with 10nM TAK-242 (EC_50_= 0.1–11nM) for 2 hours and cDNA was prepared using iSCRIPT cDNA synthesis kit (BioRad #1708891) following manufacturer’s instructions. qRT-PCR was conducted with the cycling of 5 min at 95°C for initial denaturation, 40 cycles of 30s at 95°C, 30s at 55–60°C (based on primer annealing temperatures), and 30s at 72°C followed by melting curve analysis in triplicate using SYBR Green Master Mix and a Real-time PCR system (CFX96, BioRad Laboratories, and Hercules, CA, USA). After normalizing with 18S housekeeping gene, the fold-change in mRNA expression was calculated using 2^−ΔΔCT^ method. The oligonucleotide primers used in RT-PCR (Supplementary Table 2) were purchased from Integrated DNA Technology (IDT Coralville, Iowa 52241 USA).

### 2.11 Statistical Analysis

Data are presented as the mean ± SEM. Data were analyzed using GraphPad Prism 9. The comparison between two groups for the expression of the protein of interest was performed using One-way ANOVA with Bonferroni’s post-hoc correction and Students’ t-test for statistical significance. A probability (*p*) value of < 0.05 was accepted as statistically significant.

## 3. Results

### 3.1 Angiography

**Figure 1:**
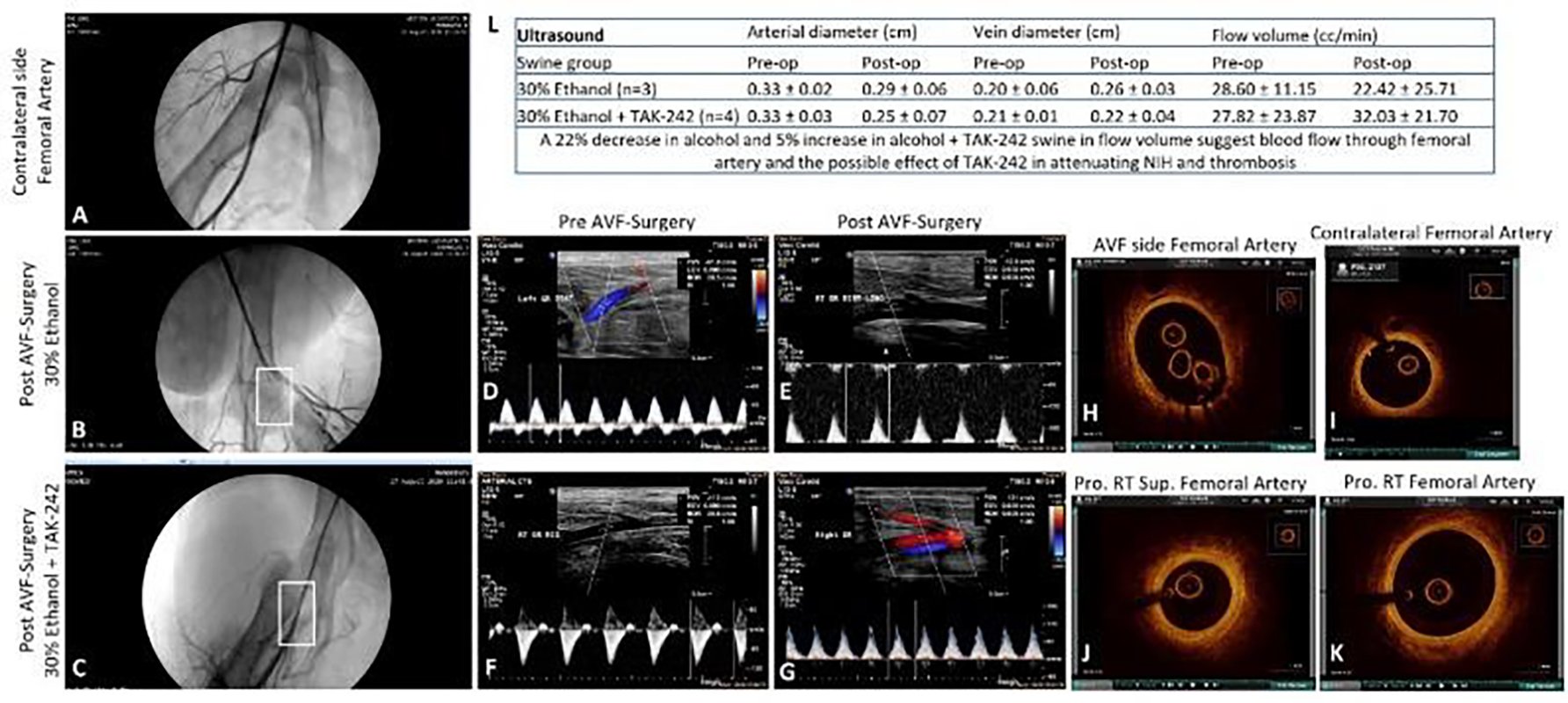
Ultrasound, angiography, and optical coherence tomography. Angiography of the contralateral femoral artery as control and pre-and post-intervention (panels A. B, and C), ultrasound (USG) of femoral artery pre-and post-intervention (panels D-G), and optical coherence tomography (OCT) images of femoral artery pre-and post-intervention (panels H, J, and K), OCT images of the contralateral femoral artery as control (panel I) in ethanol and ethanol + TAK-242 group. Panel L shows the arterial and vein diameter and arterial flow volume before and after the intervention.

Angiogram in the ethanol group showed no contrast in the superficial FA in two swine while one swine showed moderate stenosis of the distal part of the superficial femoral artery (30% of animals showed arterial patency) with no flow in the outflow vein (AVF patency in 0% animals). In the ethanol with TAK-242 group, two swine showed good and minimal arterial flow while two swine showed absent contrast in the superficial femoral artery except for short segment distal to the take-off the circumflex branch with distal flow through collateral circulation suggestive of extensive thrombosis (50% of animals (n=2) showed arterial patency). One swine showed contrast in the outflow vein suggestive of flow through the vein (AVF patency in 25% (n=1) animals). These results were supported by the findings of USG with an increased arterial flow volume by 5% in the ethanol + TAK-242 group compared to a 22% decrease in arterial flow volume in the ethanol group (Figure 1 panel L).

### 3.2 Optical Coherence Tomography (OCT)

The FA near the AVF site in all groups was evaluated by OCT. The angiography findings were supported by OCT of the swine taken before sacrificing them. In the ethanol group, guidewire could not be progressed in one swine, the femoral artery was significantly narrowed in one swine and showed the presence of calcified thrombi, and one artery showed a thickened wall with an intimal thickness of 0.10mm compared to 0.04mm of the contralateral FA. In the ethanol with TAK-242 group, one swine had normal OCT, one swine showed red thrombus, one showed white thrombus, and guidewire could not be progressed in one swine. The arteries with red and white thrombus were stenosed compared to the contralateral FA. These findings suggest the possible protective role of TLR-4 inhibition in maintaining the FA structure and patency (Figure 1).

### 3.3 Histomorphometric studies

**Figure 2:**
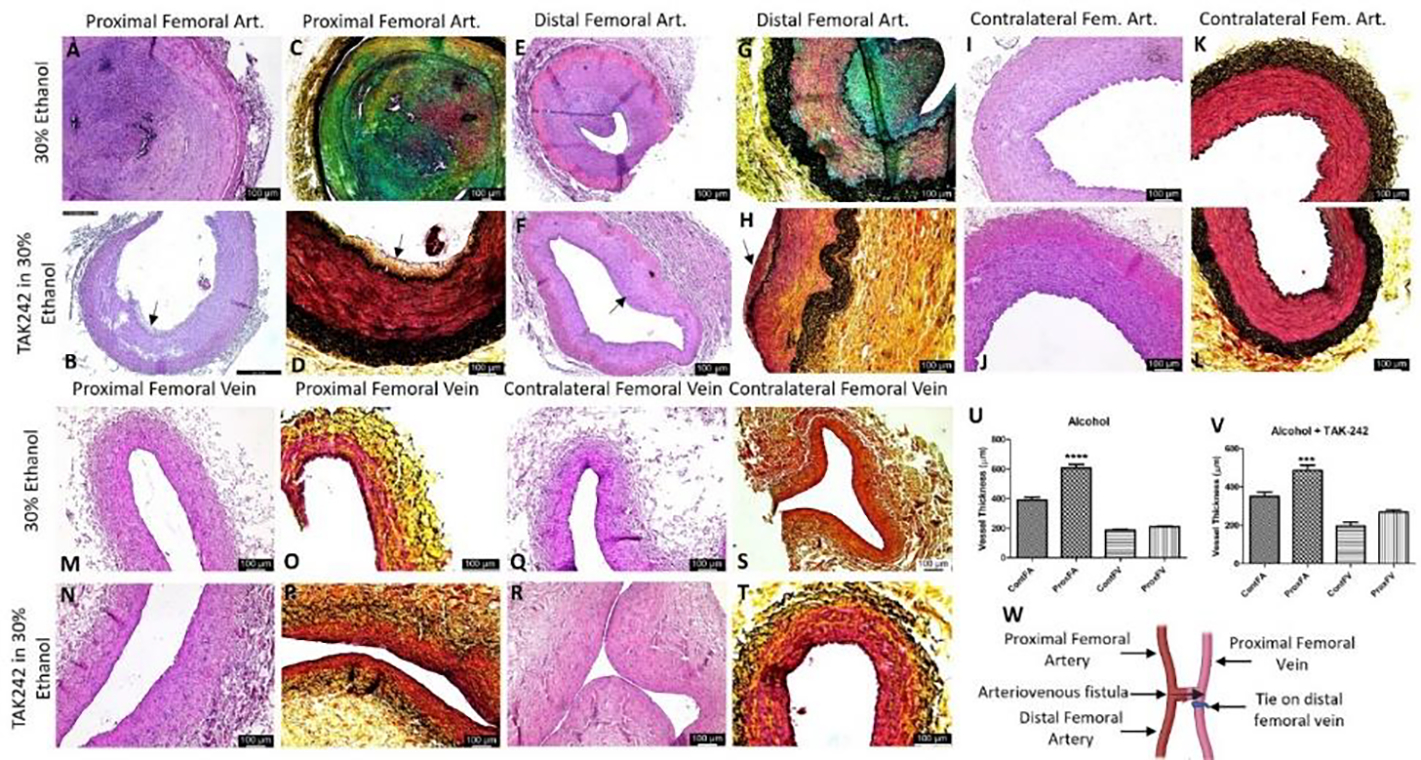
Hematoxylin and eosin and Movat-pentachrome staining of the femoral artery and femoral vein involved in the anastomosis in ethanol and ethanol + TAK-242 group and of contralateral side femoral artery and femoral vein as control. H & E staining of the femoral artery proximal to anastomosis (proximal femoral artery, panels A and B), femoral vein proximal to anastomosis (proximal femoral vein, panels M and N), and femoral artery distal to anastomosis (distal femoral artery, panels E and F); contralateral femoral artery and vein (panels I, J, Q, and R). Movat-pentachrome staining of the femoral artery proximal to anastomosis (panels C and D), femoral vein proximal to anastomosis (panels O and R), and femoral artery distal to anastomosis (panels G and H); contralateral femoral artery and vein (panels K, L, S, and T). Panel U showed vessel thickness in the ethanol group and panel V shows vessel thickness in the ethanol + TAK-242 group. Panel W depicts the representation of AVF. The arrows show the presence of neointimal hyperplasia (NIH). The data are presented as mean ± SEM (n=3 in ethanol and n=4 in ethanol + TAK-242 group). ***p<0.001 and ****p<0.0001.

The comparison of the PFA and DFA (Figure 2 panel W) revealed NIH and stenosis with a minimum opening of the lumen in the ethanol group compared to the contralateral FA. In two swine, the PFA near anastomosis was fibrosed and cannot be collected; in such cases, the FA collected at a higher level showed an open lumen with NIH. In the ethanol + TAK-242 group, PFA and DFA revealed NIH with open lumen and vessel wall thickening. The contralateral FA in the ethanol + TAK-242 group showed normal histology (Figure 2, panel J). Additionally, foci of moderate to severe inflammation with necrotic core were noted in the NIH and thrombosed area in the ethanol group while the severity of inflammation was less in TAK-242 treated group (Figure 2, panels A, E, B, and F ). The FV proximal and near the site of anastomosis (PFV) was in the fibrosed tissue and was tough to collect so the adjacent PFV was collected. H&E staining of the collected PFV showed patent lumen while the FV adjacent to AVF showed fibrosed tissue (data not shown), so to investigate the effect of the blood flow on the PFV remodeling was assessed by measuring the PFV thickness on H and E images. The thickness measurement for the PFA and PFV in H&E images revealed thickened PFA compared to contralateral FA in both groups (Figure 2 panels U and V) while thickened PFV compared to contralateral FV was only in the ethanol + TAK-242 group (Figure 2 panel V). These findings were supported by the USG findings of increased diameter of the FV at the endpoint compared to preintervention in ethanol (0.26cm vs 0.2 cm) and ethanol + TAK-242 (0.22cm vs 0.21cm) group (Figure 1 panel L). Movat Pentachrome staining of PFA revealed increased collagen (yellow), fibrin (bright red), glycans (blue), and elastin (black/blue-black) staining in NIH and atherosclerotic plaque area compared to the contralateral FA. The subjective scoring (based on Supplementary Table 3) showed weak yellow staining (score of 2) in the ethanol group while the ethanol + TAK-242 group showed a very weak yellow stain (score of 1). The staining for glycans deposition showed weak to moderate blue staining (score of 2–3) in the ethanol group, and no staining in the ethanol + TAK-242 group. Fibrin deposition (very weak to weak intense red, score of 1–2) was present in the ethanol group while ethanol + TAK-242 group swine showed no staining for fibrin. The elastin staining was increased in the medial layer (weak dark purple to black staining) of PFA for both groups compared to the contralateral FA. The collagen staining was present in both PFV and contralateral FV but was more in PFV compared to contralateral FV (Figure 2 panels C, D, G, H, O, P, S, and T). Compared to the contralateral FA, medial elastin was disorganized in both the ethanol and TAK-242 group and disorganization was more evident in the ethanol + TAK-242 group (Figure 2 panels D and H) compared to the ethanol group (Figure 2 panels C and E) where arteries were thrombosed.

### 3.4 Immunostaining

**Figure 3:**
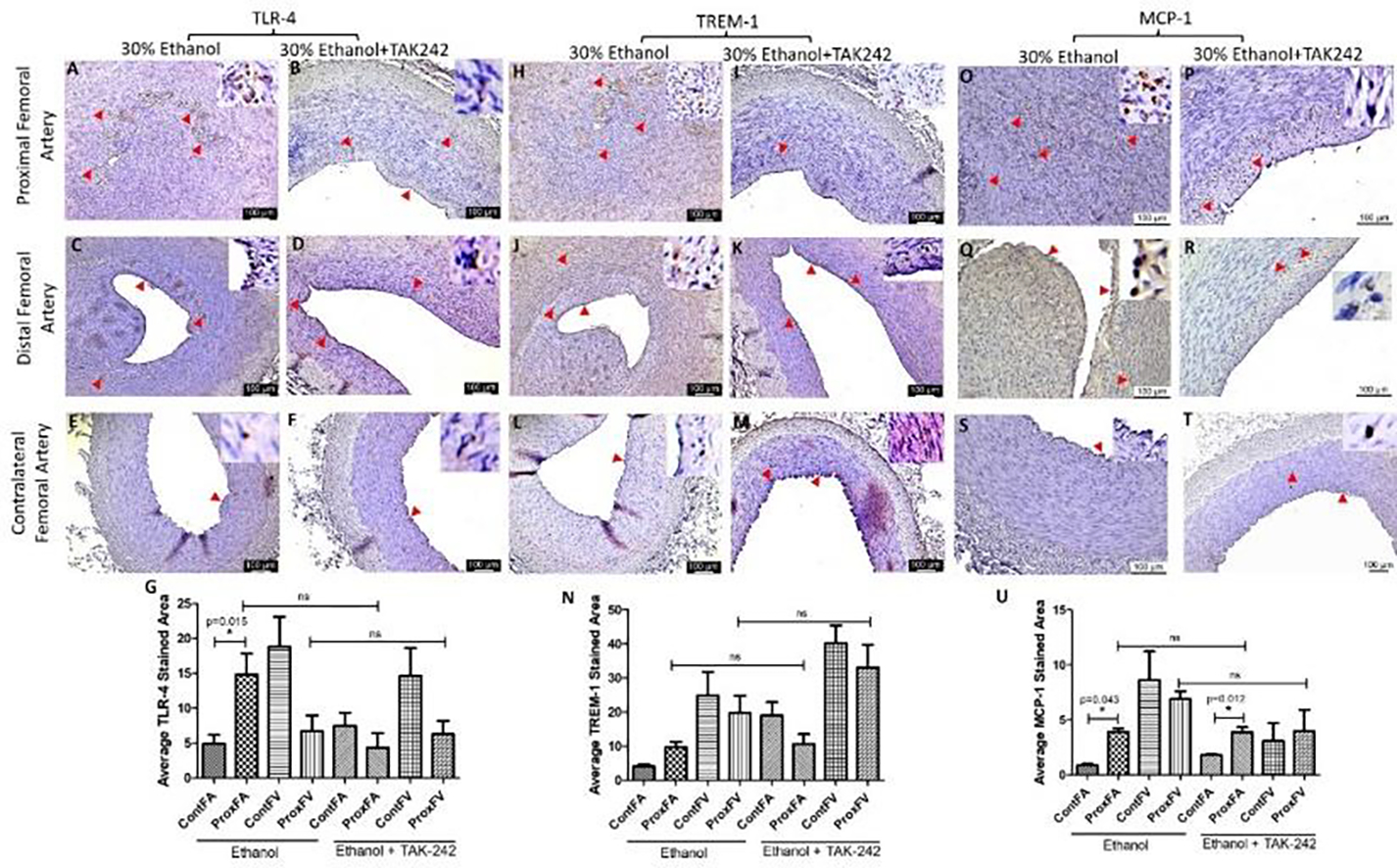
Immunohistochemistry for toll-like receptor (TLR)-4, triggering receptor expressed on myeloid cells (TREM)-1, and monocyte chemoattractant protein (MCP)-1 in the femoral artery in ethanol and ethanol + TAK-242 group. TLR-4 staining (panels A to F), TREM-1 staining (panel H to M), and MCP-1 staining (panels O to T) in the proximal femoral artery, distal femoral artery, and contralateral femoral artery. Average stained intensity for TLR-4 (panel G), TREM-1 (panel N), and MCP-1 (panel U). The red arrowheads show positively stained cells and inserts show high magnification images. There are representative images from all animals in the study. Data are presented as mean ± SEM (n=3 in ethanol and n=4 in ethanol + TAK-242).

**Figure 4:**
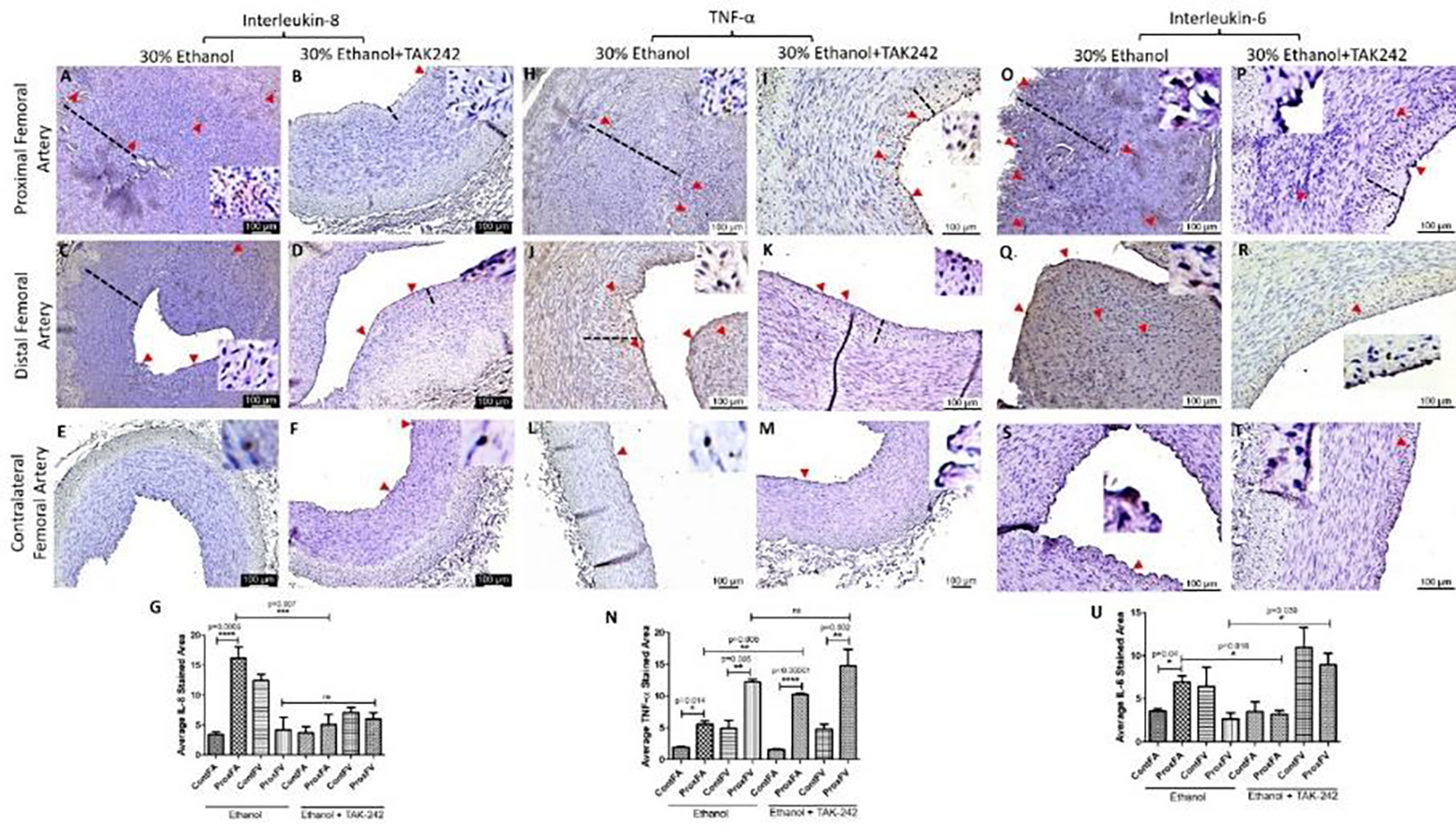
Immunohistochemistry for interleukin (IL)-8, tumor necrosis factor (TNF)-α, and IL-6 in the femoral artery in ethanol and ethanol + TAK-242 group. IL-8 staining (panels A to F), TNF-α staining (panels H to M), and IL-6 staining (panels O to T) in the proximal femoral artery, distal femoral artery, and contralateral femoral artery. Average stained intensity for IL-8 (panel G), TNF- α (panel N), and IL-6 (panel U). The red arrowheads show positively stained cells and inserts show high magnification images. There are representative images from all animals in the study. Data are presented as mean ± SEM (n=3 in ethanol and n=4 in ethanol + TAK-242).*p<0.05, **p<0.01, ***p<0.001, ****p<0.0001.

IHC of the FA revealed increased immunopositivity for the mediators of inflammation including TLR-4, TREM-1, MCP-1, IL-8, TNF-α, and IL-6 (Figures 3 and 4) in ethanol and ethanol + TAK-242 group compared to the contralateral FA. The subjective evaluation of immunostaining showed increased expression of TLR-4 and TREM-1 in PFA in the ethanol group compared to the ethanol + TAK-242 group (Figure 3 panels G and N). TLR-4 and TREM-1 expression were higher in PFA in the ethanol group while lower in the ethanol + TAK-242 group compared to the contralateral FA. Immunopositivity for MCP-1 was higher in PFA in both ethanol and ethanol + TAK-242 group (Figure 3 panel U). The immunopositivity for TLR-4, TREM-1, and MCP-1 was lower in PFV in the ethanol and TAK-242 group except that MCP-1 expression was higher in PFV in the TAK-242 group compared to the contralateral artery (Figure 3 panels G, N, and U; Supplementary Figure 1).

The protein expression for IL-8, TNF-α, and IL-6 was higher in PFA in both ethanol and TAK-242 treated group compared to contralateral FA except that the expression of IL-6 in PFA and the contralateral FA was comparable in the TAK-242 group (Figure 4 panels G, N, and U). In PFA; IL-8 expression was higher in the ethanol group compared to the ethanol + TAK-242 group; TNF-α expression was higher in the ethanol + TAK-242 group compared to ethanol group, and IL-6 expression was higher in the ethanol group compared to ethanol + TAK-242 group. The immunopositivity for IL-8 and IL-6 was lower while immunopositivity for TNF- α was higher in PFV compared to the contralateral FV in the ethanol group. The immunopositivity for IL-8 and IL-6 was lower while the immunopositivity for TNF- α was higher in PFV compared to contralateral FV in ethanol + TAK-242 group (Figure 4 panels G, N, and U; Supplementary Figure 2).

**Figure 5:**
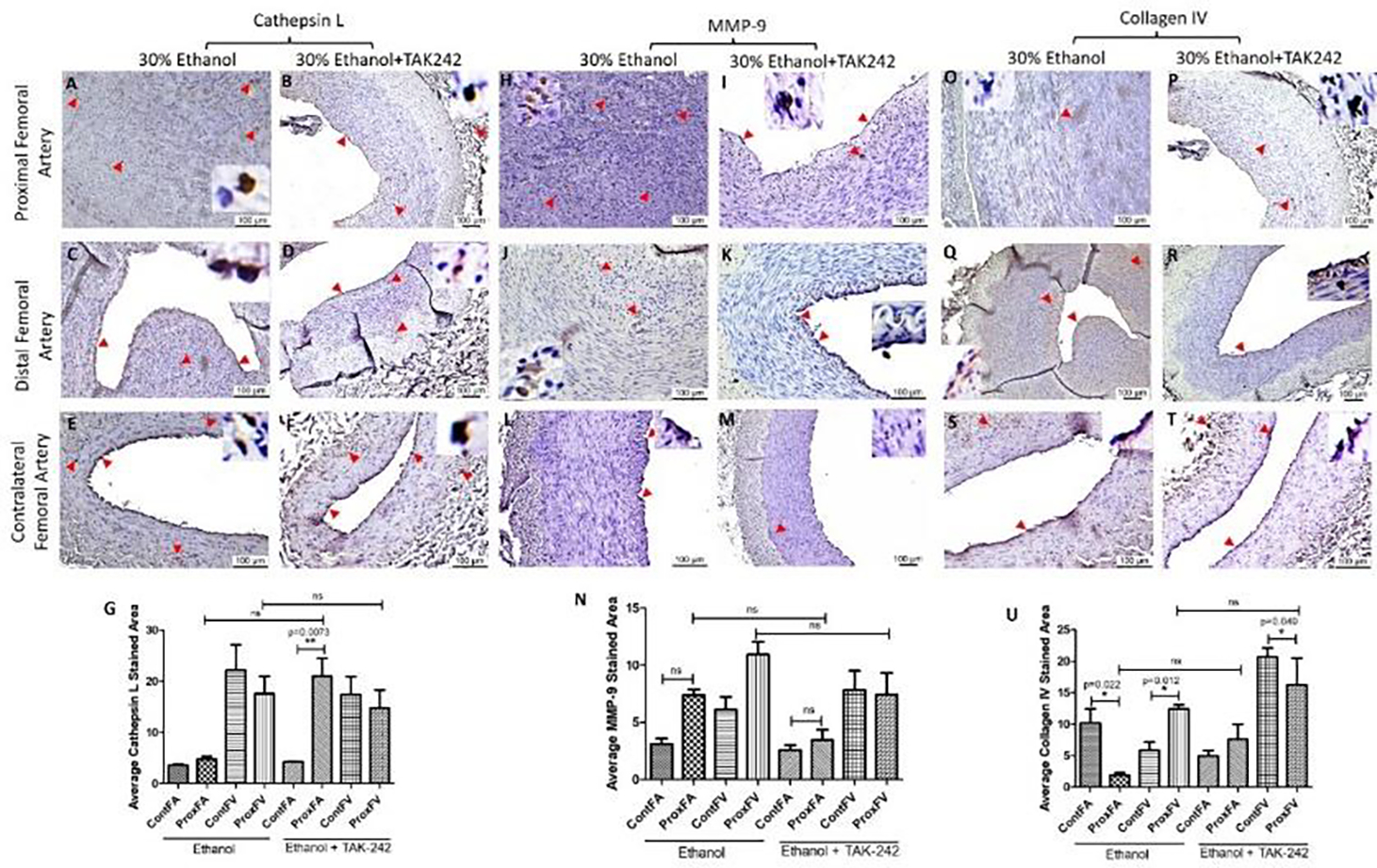
Immunohistochemistry for cathepsin L, matrix metalloproteinases (MMP)-9, and collagen IV in the femoral artery in ethanol and ethanol + TAK-242 group. Cathepsin L staining (panels A to F), MMP-9 staining (panel H to M), and collagen IV staining (panels O to T) in the proximal femoral artery, distal femoral artery, and contralateral femoral artery. Average stained intensity for cathepsin L (panel G), MMP-9 (panel M), and collagen IV (panel U). The red arrowheads show positively stained cells and inserts show high magnification images. There are representative images from all animals in the study. Data are presented as mean ± SEM (n=3 in ethanol and n=4 in ethanol + TAK-242).*p<0.05.

The expression for the mediators of collagen degradation cathepsin L and MMP-9 was higher in PFA compared to the contralateral FA in both ethanol and ethanol + TAK-242 group (Figure 5). In PFA, Cathepsin L expression was higher in ethanol + TAK-242 compared to the ethanol group while MMP-9 expression was higher in ethanol compared to ethanol + TAK-242 group (Figure 5 panels G and N). Collagen IV expression in PFA was lower in ethanol while higher in the ethanol + TAK-242 group compared to the contralateral FA (Figure 5 panel U). In PFV, compared to the contralateral FA, the expression of cathepsin L was comparable in ethanol and lower in the ethanol + TAK-242 group, the expression of MMP-9 was higher in PFV in ethanol and lower in the ethanol + TAK-242 group, and collagen IV expression was higher in ethanol + TAK-242 and lower in ethanol group (Figure 5 panels G, N, and U; Supplementary Figure 3). In PFV, Cathepsin L, MMP-9, and collagen IV expression were higher in ethanol compared to the ethanol + TAK-242 group.

The expression of α-SMA in PFA was higher in ethanol and lower in the ethanol + TAK-242 group compared to the contralateral FA (Supplementary Figure 4) while the expression of α-SMA in PFV was lower in ethanol and higher in ethanol + TAK-242 group compared to the contralateral FV (Supplementary Figure 4 panel G; Supplementary Figure 5). The expression of α-SMA in PFA was higher in ethanol compared to ethanol + TAK-242 group while the expression of α-SMA in PFV was higher in ethanol + TAK-242 compared to the ethanol group. Vimentin expression in PFA in both ethanol and ethanol + TAK-242 group was higher compared to the contralateral FA and the expression in PFV was lower in both groups compared to the contralateral FV. The expression of vimentin in PFA and PFV was comparable in both ethanol and ethanol + TAK-242 group (Supplementary Figure 4 panel N; Supplementary Figure 5).

The expression of growth factors and cell adhesion molecules TGF-β and VCAM-1 responsible for ECM deposition, fibroblast transition, and thrombosis was higher in PFA compared to the contralateral FA in both ethanol and ethanol + TAK-242 group (Supplementary Figure 6 panels G and N). The immunopositivity for TGF-β in PFV was higher in ethanol while lower in the ethanol + TAK-242 group compared to the contralateral FV. The expression of VCAM-1 in PFV compared to contralateral femoral vein was lower in ethanol and ethanol + TAK-242 group (Supplementary Figure 5 and Supplementary Figure 6 panels G and N). The expression for TGF-β was comparable while the expression for VCAM-1 was lower in the ethanol group compared to ethanol + TAK-242 in PFA. The expression for TGF-β was higher while the expression for VCAM-1 was lower in the ethanol group compared to ethanol + TAK-242 in PFV (Supplementary Figure 5 and Supplementary Figure 6 panels G and N).

The expression for these proteins was mainly in the NIH and thrombosed area in the PFA in both groups and intimal layer in PFV and contralateral FA and FV. The expression of TLR-4, VCAM-1, cathepsin L, and MMP-9 was present in the intima, intimal-medial junction, and medial layer of the PFA. The expression for IL-8, IL-6, TNF-α, TLR-4, TREM-1, MCP-1, TGF-β, VCAM-1, cathepsin L, MMP-9, collagen IV, α-SMA, and vimentin in contralateral FA was in the intima while in the intima and media of the PFV and contralateral FV. The expression of IL-6, IL-8, TLR-4, MMP-9, and α-SMA in PFA was higher in the ethanol group compared to the ethanol + TAK-242 group while the expression of collagen IV, cathepsin L, and VCAM-1 was higher in the ethanol + TAK-242 group. The expression of TGF-β, MMP-9, and MCP-1 was higher in the ethanol group and the expression of α-SMA, VCAM-1, collagen IV, IL-6, and IL-8 were higher in the ethanol + TAK-242 group in PFV (Supplementary Table 4).

### 3.5 Real-Time Polymerase Chain Reaction

**Figure 6:**
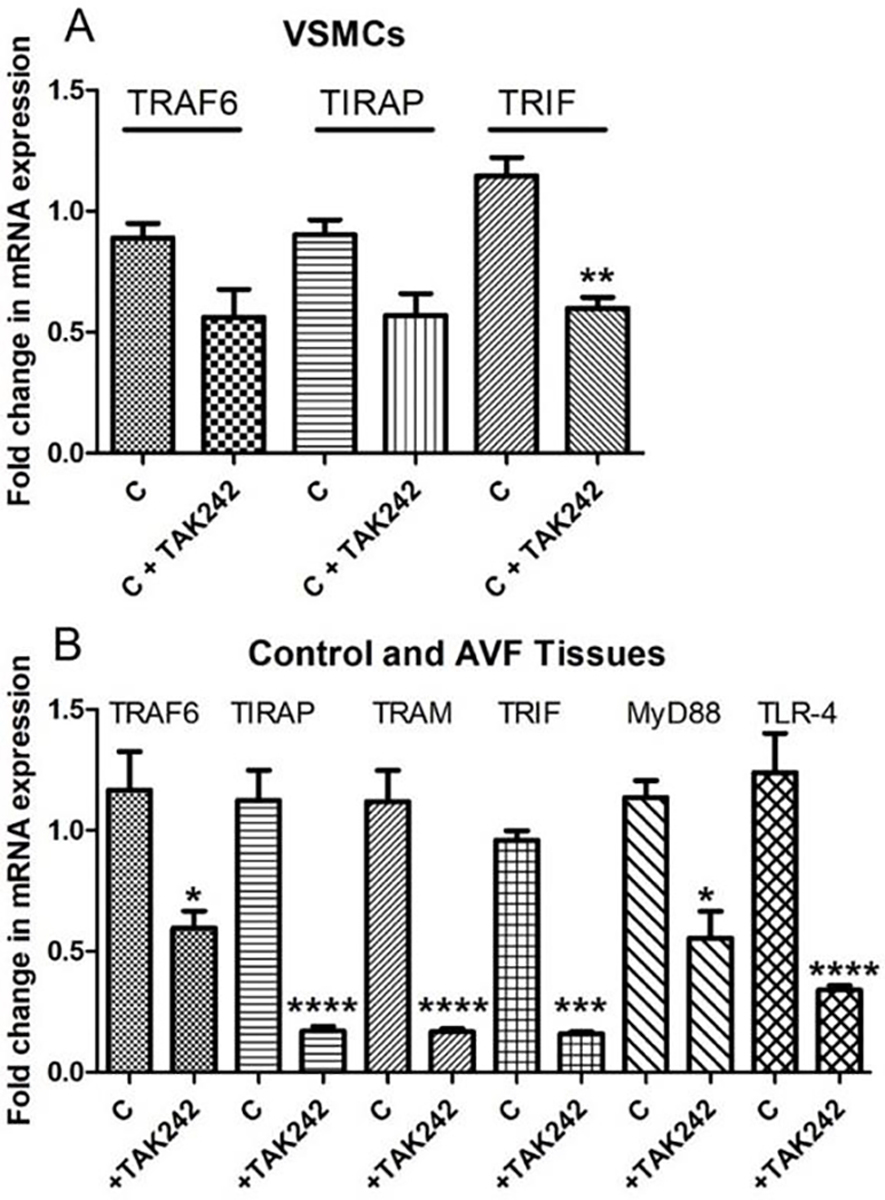
RT-PCR for mRNA expression of TLR-4 downstream signaling and the effect of TAK-242. a) effect of TAK-242 on TNF Receptor Associated Factor 6 (TRAF6), TIR Domain Containing Adaptor Protein (TIRAP), and TIR-domain-containing adapter-inducing interferon-β (TRIF) in VSMCs and the effect of TAK-242 on TNF Receptor Associated Factor 6 (TRAF6), TIR Domain Containing Adaptor Protein (TIRAP), TIR-domain-containing adapter-inducing interferon-β (TRIF), TRIF-related adaptor molecule (TRAM), myeloid differentiation primary response 88 (MyD88), and toll-like receptor (TLR)-4 in in-vivo. Data are presented as mean ± SD.*p<0.05, **p<0.01, ***p<0.001, ****p<0.0001.

qRT-PCR revealed attenuation of TRAF6, TIRAP, and TRIF mRNA expression in VSMCs treated with TAK-242 compared to untreated VSMCs. The results were not suggestive of any change in mRNA expression of TRAM, MyD88, and TLR-4 in VSMCs with TAK-242 treatment (Figure 6A). However, the qRT-PCR analysis of femoral arteries involved in AVF, and contralateral side arteries revealed attenuation of TRAF6, TIRAP, TRIF, TRAM, MyD88, and TLR-4 in femoral artery involved in AVF and treated with TAK-242 compared to the contralateral FA (Figure 6B).

## 4. Discussion

The ultrasound sonography of the FA showed a 5% increase in arterial flow volume after 12 weeks in the TAK-242 group compared to a 22% decrease in the ethanol alone group. The protective effect of TAK-242 in attenuating thrombus formation was also revealed by angiography where 25% of animals showed contrast in outflow vein compared to moderate stenosis of the distal part of the superficial femoral artery with contrast in only 0% of animals in the ethanol group. OCT findings also supported the effect of TAK-242 and OCT was normal in 25% animals, 50% animals with small thrombus formation, and 25% with a stenosed vessel in the TAK-242 group compared to ethanol alone group where the femoral artery was significantly narrowed and thrombosed (Figure 1). These results suggest the effectiveness of inhibiting TLR-4 by TAK-242 in attenuating vessel stenosis and maintaining the patency of the FA involved in AVF and minimal to moderate flow through the fistula at the end of 12 weeks. Ethanol reduces the neointima formation and stenosis and preserves the arterial lumen following balloon injury in part by decreasing LDL oxidation [28] and the results of this study with 33% FA with partially patent lumen are per the previous reports. Further, increased patency of the FA in the TAK-242 group is suggestive of the additive effect of inhibiting TLR-4 in maintaining arterial patency and the effect of TAK-242 in attenuating NIH and thrombosis [24].

Significant luminal stenosis of the FA and FV due to eccentric NIH and intima-media thickening is central to AVF failure [6, 29] and initial aggressive treatment may salvage the AVF [30]. NIH and stenosis with a minimal luminal opening of PFA and DFA in the ethanol group while an open lumen with minimal NIH in the TAK-242 group on histomorphometric studies (Figure 2) is suggestive of the effect of TLR-4 inhibition in attenuating inflammation, NIH, and stenosis. Further, thickened PFA compared to contralateral FA in both groups and thickened PFV compared to contralateral FV in the TAK-242 group only is suggestive of vessel remodeling due to increased blood flow through AVF at 12-week time point and also indicate reduced thrombosis and patent femoral artery in TAK-242 group (Figure 2). Vessels remodeling in AVF is supported by an increased femoral vein diameter on USG (Figure 1). Outflow vein remodeling with vessel wall thickening due to shear stress and hemodynamic changes after AVF creation is associated with AVF maturation [31] and vessel wall thickening in this study is indicative of the beneficial effect of TLR-4 inhibition.

Increased expression of TLR-4 and upregulation of its ligands stimulate atherosclerosis [32]. Excessive expression of TLR-4 is associated with excessive NIH and stenosis leading to AVF failure and increased expression of TLR-4 in the ethanol group and decreased expression in the TAK-242 group indicates the efficacy of TAK-242. Further, decreased expression of TLR-4 and patent PFA, PFV, and DFA in the TAK-242 group is indicative of the effectiveness of TAK-242 in attenuating thrombosis and stenosis, however, arterial patency in only 50% and flow through AVF in 25% of animals warrant further research with an increased number of animals. The anti-inflammatory and inhibitory effect of TAK-242 on TLR-4 is supported by the decreased mRNA expression of TRAF6, TIRAP, TRIF, TRAM, MyD88, and TLR-4 in FA involved in AVF with RT-PCR as well as by attenuated expression of TRAF6, TIRAP, and TRIF in VSMCs treated with TAK-242 (Figure 6). The different in-vitro and in-vivo effects of TAK-242 may be context-dependent (different treatment and types of tissues, one in VSMCs and the other in animals) [33–36].

Chronic inflammation, and progressive NIH, and atherosclerosis are associated with vessel thrombosis and stenosis, and increased expression of inflammatory mediators TREM-1, regulated by TLR-4 activation [9, 18, 23, 37, 38], might play a role in sterile inflammation in AVF maturation failure and patency of AVF [39]. Thus attenuating TREM-1 expression may attenuate inflammation and vessel stenosis and a decreased TREM-1 expression in the TAK-242 group compared to the ethanol group (9.62 ±1.42 vs 10.62 ± 3.40) suggest that TAK-242 not only decreases TLR-4 expression but also TREM-1 though not significant (Figure 3). TLR-4 and TREM-1 are expressed mainly on macrophages and these cells are recruited during acute inflammation to clear off the debris, but sustained recruitment of these immune cells may lead to chronic inflammation [23, 26, 40, 41]. The recruitment of immune cells is facilitated by MCP-1 and proinflammatory cytokines including IL-6, IL-8, and TNF-α [42]. Increased expression of MCP-1, IL-6, IL-8, and TNF-α (Figure 4) in PFA in NIH and stenosed area in the ethanol group is suggestive of inflammation [10, 31] whereas an attenuated expression of these proteins and patent lumen in TAK-242 swine indicate the beneficial effect of inhibiting TLR-4 by TAK-242 in attenuating inflammation [43]. An attenuated inflammation and thrombosis of the FA involved in AVF will favor vessel remodeling causing increased blood flow through the FV to withstand the increased blood flow, pressure, and hemodynamic changes. The patency in 50% PFA and blood flow in 25% AVF is indicative of vessel remodeling in the TAK-242 group.

Vascular wall remodeling is associated with both ECM deposition and VSMCs proliferation and migration. ECM deposition and remodeling with collagen and elastin degradation correlates with AVF maturation [31]. Increased elastin stain in the media of the PFA and PFV and collagen deposition in the NIH area suggests ongoing ECM remodeling in the TAK-242 group (Figure 2). Increased elastin, collagen, glycans, and fibrin deposition on Movat-pentachrome stain in NIH and thrombosed area of PFA in ethanol group is suggestive of remodeling but the obliteration of the lumen might be due to other factors/proteins regulating ECM remodeling including TGF-β, MMP-9, collagen IV, VSMCs phenotype, VCAM-1, and cathepsin L and the presence of inflammation due to surgical intervention as well as a systemic immune response [31]. Further, increased elastin degradation and collagen deposition are the features of vessel wall fibrosis, and this may be another factor contributing to the failure of remodeling and AVF thrombosis or non-maturation [44].

Venous adaptation to arterial flow and venous wall integrity after AVF creation pertains to the adventitial myofibroblast [45]. The presence of α-SMA and vimentin-positive (Supp. Figure 4) and desmin negative (data not shown) VSMCs in NIH, thrombosed area, and adventitia of PFA in both groups with increased positivity in FA involved in anastomosis compared to contralateral FA supports vessel remodeling [29]. Further, an increased presence of α-SMA and vimentin-positive cells in the PFV in the TAK-242 group compared to ethanol and contralateral FV suggest the presence of proliferating VSMCs and myofibroblasts in remodeling vessels. An open lumen in both PFA and PFV in the TAK-242 group is suggestive of favorable vessel remodeling and the potential of inhibiting TLR-4. ECM remodeling, involved in vessel remodeling, is regulated by TGF-β and VCAM-1 [31]. The expression of TGF-β increases after AVF creation [46] and higher expression of VCAM-1 is associated with thrombosis and stenotic AVF [47]. An increased TGF-β and VCAM-1 expression in PFA compared to contralateral FA with a higher expression in the ethanol group compared to the TAK-242 group (Supp. Figure 6) suggest the role of inhibiting TLR-4 in preventing vessel stenosis and thrombosis. These findings support the necropsy findings of fibrosed and thrombosed femoral vessels near the anastomosis site in the ethanol group and a lower expression of these proteins at 12-week in the TAK-242 group suggests the role of inhibiting TLR-4 in favorable vessel remodeling.

This notion of favorable vessel remodeling via ECM remodeling with TLR-4 inhibition is supported by an increased MMP-9 and cathepsin L expression and decreased collagen expression in PFA in both groups (Figure 5) and increased collagen in PFV (Supp. Figure 3) [17]. The vascular adaptive response includes the formation of ECM components, MMP secretion, and collagen deposition to strengthen the fistula wall [17], however, excessive collagen deposition is associated with fistula nonmaturation, stenosis, and fibrosis [48]. Increased collagen expression in PFV in the ethanol group and decreased expression in the TAK-242 group are suggestive of favorable remodeling in the TAK-242 group. These results are supported by increased deposition of collagen, fibrin, glycans, and elastin in the ethanol group compared to the TAK-242 group (Figure 2). MMPs regulating proliferation and migration of VSMCs play a crucial role in intimal thickening, atherosclerosis, and thrombosis [15]. MMP expression is associated with matrix degradation and expression levels of TGF-β, whose expression is increased in early as well as late AVF, regulates matrix remodeling by ECM deposition [17]. Proinflammatory cytokines including IL-6, IL-8, and TNF-α regulate the expression of MMPs [9, 40, 41]; thus, targeting inflammatory mediators is of interest to modify the anastomosis microenvironment favorable for AVF maturation. An attenuated expression of mediators of inflammation with patent lumen and presence of flow in outflow vein in TAK-242 group is suggestive of the beneficial role of inhibiting TLR-4 in attenuating early thrombosis playing a critical role in AVF nonmaturation.

**Figure 7:**
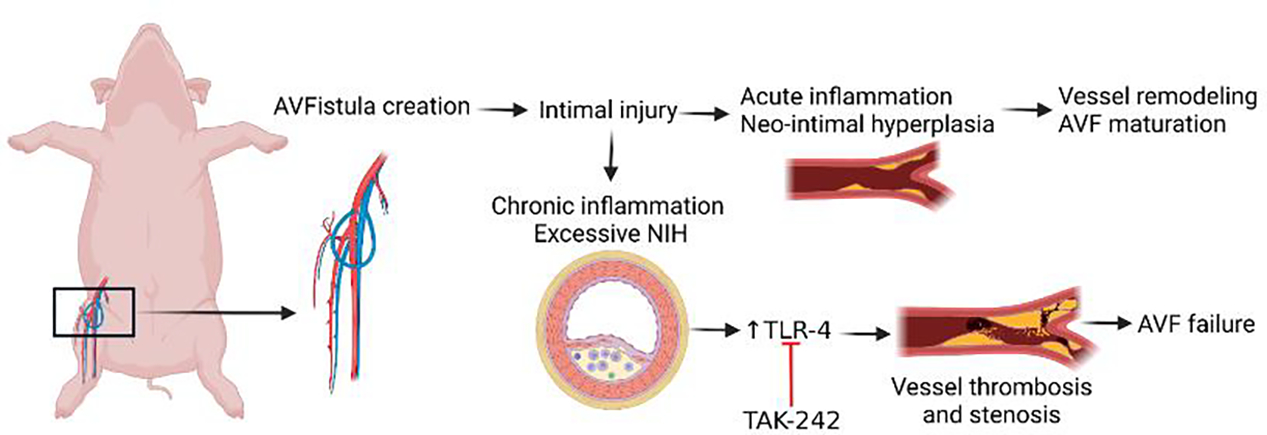
Schematic representation of the study and the therapeutic role of TLR-4 inhibition. Chronic inflammation and increased TLR-4 expression play an important role in the pathogenesis of early vessel thrombosis and stenosis after AVF creation and inhibition of TLR-4 with TLR-4 inhibitors may enhance AVF maturation.

Collectively, the results of this study suggest that targeting TLR-4 to attenuate inflammation and early thrombosis (Figure 7 and Supp. Figure 7) may have therapeutic potential, however, studies with an increased number of animals are warranted.

## 5. Conclusion

AVF non-maturation is associated with excessive NIH, thrombosis, and stenosis of the vasculature, and AVF maturation is associated with the arterial and venous outward remodeling with flow through the outflow vein. Early thrombosis is critical for AVF failure and targeting inflammation to attenuate stenosis and thrombosis has therapeutic potential to keep femoral artery patent and increase blood flow through AVF.

### Limitations of the Study

This study revealed that TAK-242 targeting TLR-4 attenuates early thrombosis and makes femoral vessels patent. Although a small number of animals is a limitation of this study, nonetheless the findings are suggestive of the potency of targeting TLR-4 in maintaining lumen patency and blood flow through AVF via attenuating inflammation and facilitating ECM remodeling. Other limitation could be the use of ethanol as the vehicle for TAK-242. Therefore, other clinically-relevant vehicle must be examined for potentially a better efficacy of TAK-242. Additionally, adventitial fibrosis may be an impeding factor for vessel remodeling and AVF maturation thus promoting wall thickening of outflow vein strength without exuberant thickening, NIH, and adventitial fibrosis should be the focus of research in addition to targeting inflammation. Another limitation was the fibrosed femoral arteries and veins near the site of anastomosis and this may be animal model-dependent and need sample collections more incisively. Further, investigation of additional factors like coagulation status, the role of immune cells, VSMCs phenotypic changes, endothelial dysfunction, and targeting adventitia is of utmost importance to design better therapeutics and strategies of AVF maturation.

## Supplementary Material

1 Figure S1: Immunohistochemistry for toll-like receptor (TLR)-4, triggering receptor expressed on myeloid cells (TREM)-1, and monocyte chemoattractant protein (MCP)-1 in the femoral vein in ethanol and ethanol + TAK-242 group. TLR-4 staining (panels A to D), TREM-1 staining (panel E to H), and MCP-1 staining (panels I to L) in the proximal femoral vein and contralateral femoral vein. The red arrowheads show positively stained cells and inserts show images in higher magnification. There are representative images from all animals in the study.
Figure S2: Immunohistochemistry for interleukin (IL)-8, tumor necrosis factor (TNF)-α, and IL-6 in the femoral vein in ethanol and ethanol + TAK-242 group. IL-8 staining (panels A to D), TNF-α staining (panel E to H), and IL-6 staining (panels I to L) in the proximal femoral vein and contralateral femoral vein. The red arrowheads show positively stained cells and inserts show images in higher magnification. There are representative images from all animals in the study.
Figure S3: Immunohistochemistry for cathepsin L, matrix metalloproteinases (MMP)-9, and collagen IV in the femoral vein in ethanol and ethanol + TAK-242 group. Cathepsin L staining (panels A to D), MMP-9 staining (panel E to H), and collagen IV staining (panels I to L) in the proximal femoral vein and contralateral femoral vein. The red arrowheads show positively stained cells and inserts show images in higher magnification. There are representative images from all animals in the study.
Figure S4: Immunohistochemistry for alpha-smooth muscle action (α-SMA) and vimentin in the femoral artery in ethanol and ethanol + TAK-242 group. α-SMA staining (panels A to F) and vimentin staining (panel H to M) in the proximal femoral artery, distal femoral artery, and contralateral femoral artery. Average stained intensity for α-SMA (panel G) and vimentin (panel N). The red arrowheads show positively stained cells and inserts show images in higher magnification. There are representative images from all animals in the study. Data are presented as mean ± SEM (n=3 in ethanol and n=4 in ethanol + TAK-242). *p<0.05, **p<0.01 ****p<0.0001.
Figure S5: Immunohistochemistry for alpha-smooth muscle action (α-SMA), vimentin, transforming growth factor-beta (TGF-β), and vascular cell adhesion molecule (VCAM)-1 in the femoral vein in ethanol and ethanol + TAK-242 group. α-SMA staining (panels A to D) and vimentin staining (panels E to H), TGF-β staining (panels I to L), and VCAM-1 staining (panels M to P) in the proximal femoral vein and contralateral femoral vein. The red arrowheads show positively stained cells and inserts show images in higher magnification. There are representative images from all animals in the study.
Figure S6: Immunohistochemistry for transforming growth factor-beta (TGF-β) and vascular cell adhesion molecule (VCAM)-1 in the femoral artery in ethanol and ethanol + TAK-242 group. TGF-β staining (panels A to F) and VCAM-1 staining (panel H to M) in the proximal femoral artery, distal femoral artery, and contralateral femoral artery. Average stained intensity for TGF-β (panel G) and VCAM-1 (panel N). The red arrowheads show positively stained cells and inserts show images in higher magnification. There are representative images from all animals in the study. Data are presented as mean ± SEM (n=3 in ethanol and n=4 in ethanol + TAK-242).*p<0.05, **p<0.01.
Figure S7: (i) Full area sections of the proximal femoral artery (panels A and B), distal femoral artery (panels E and F), proximal femoral vein (panels G and H), contralateral femoral artery (panels C and D), and vein (panels I and J) in ethanol and ethanol + TAK-242 group. There are representative images from all animals in the study. (ii) Flow schematics of the study hypothesis.
Table S1: Primary antibodies used in immunostaining.
Table S2: Nucleotide sequence of the genes used in RT-PCR.
Table S3: Criteria used to score Movat Pentachrome staining.
Table S4: Summary of the expression profile of the protein of interest in the femoral artery and femoral vein on AVF and contralateral side. PA- femoral artery proximal to the site of anastomosis, PV- femoral vein proximal to the site of anastomosis, CA- contralateral femoral artery, CV- contralateral femoral vein, ~comparable or approximately equal, < less than, > more than. AVF: Arteriovenous fistula, IL: interleukin, MCP-1: monocyte chemotactic protein 1, MMP: matrix metalloproteinases, α-SMA: alpha-smooth muscle actin, TGF- β: transforming growth factor-beta, TNF-α: tumor necrosis factor-alpha, TREM-1: triggering receptor expressed on myeloid cells-1, TLR-4: toll-like receptor-4, VCAM-1: vascular cell adhesion molecule 1.

## Data Availability

All the analyzed data have been provided in the manuscript and Supplementary Files. The datasets (raw data) used for analysis during the current study will be made available by the corresponding author upon request.
