# Supplementary material for: TLR-4 Inhibition Attenuates Inflammation, Thrombosis, and Stenosis in Arteriovenous Fistula in Yucatan Miniswine
Source: Cardiol Cardiovasc Med. Author manuscript; Available in PMC 2022 Sep 21. (PMC9491704; doi:10.26502/fccm.92920280)
Supplement: 1 — Figure S1: Immunohistochemistry for toll-like receptor (TLR)-4, triggering receptor expressed on myeloid cells (TREM)-1, and monocyte chemoattractant protein (MCP)-1 in the femoral vein in ethanol and ethanol + TAK-242 group. TLR-4 staining (panels A to D), TREM-1 staining (panel E to H), and MCP-1 staining (panels I to L) in the proximal femoral vein and contralateral femoral vein. The red arrowheads show positively stained cells and inserts show images in higher magnification. There are representative images from all animals in the study. Figure S2: Immunohistochemistry for interleukin (IL)-8, tumor necrosis factor (TNF)-α, and IL-6 in the femoral vein in ethanol and ethanol + TAK-242 group. IL-8 staining (panels A to D), TNF-α staining (panel E to H), and IL-6 staining (panels I to L) in the proximal femoral vein and contralateral femoral vein. The red arrowheads show positively stained cells and inserts show images in higher magnification. There are representative images from all animals in the study. Figure S3: Immunohistochemistry for cathepsin L, matrix metalloproteinases (MMP)-9, and collagen IV in the femoral vein in ethanol and ethanol + TAK-242 group. Cathepsin L staining (panels A to D), MMP-9 staining (panel E to H), and collagen IV staining (panels I to L) in the proximal femoral vein and contralateral femoral vein. The red arrowheads show positively stained cells and inserts show images in higher magnification. There are representative images from all animals in the study. Figure S4: Immunohistochemistry for alpha-smooth muscle action (α-SMA) and vimentin in the femoral artery in ethanol and ethanol + TAK-242 group. α-SMA staining (panels A to F) and vimentin staining (panel H to M) in the proximal femoral artery, distal femoral artery, and contralateral femoral artery. Average stained intensity for α-SMA (panel G) and vimentin (panel N). The red arrowheads show positively stained cells and inserts show images in higher magnification. There are repres [file NIHMS1834865-supplement-1.pdf]

Supplementary Data

Antibody	Catalog	Dilution
Rabbit anti-TLR4	ab213676	1:100
Rabbit anti-TREM-1	ab-200729	1:100
Rabbit anti-TNF-α	ab6671	1:200
Rabbit anti-IL8	ab7747	1:200
Rabbit anti-cathepsin L	LS-C293267	1:200
Rabbit anti-collagen IV	ab6586	1:100
Rabbit anti-TGF-β	sc-146	1:50
Mouse anti-vimentin	sc-6260	1:50
Rabbit anti-desmin	sc-14026	1:50
Rabbit anti-IL-6	ab6672	1:100
Rabbit anti-VCAM1	ab106777	1:100
Rabbit anti-MMP-9	ab38898	1:100
Rabbit anti-MCP1	ab9669	1:100
Rabbit anti-αSMA	ab5694	1:100

Table S1: Primary Antibodies Used in Immunostaining.

Gene name	Forward primer	Reverse primer
TRAF6	5'-ATGCATCTGGACGCCCTAAG-3'	5'-CCCGAGTCTGTACTTCGTGG-3'
TIRAP	5'-CCCTTCCCACAACACCAAGA-3'	5'-ACCAGGGCTTATCACTGTGC-3'
TRIF	5'-AAGGTGGCCAACACCTTCAA-3'	5'-CTTCTCCAACTGCGTCTGGT-3'
TRAM	5'-TCTTTGCTGAGATGCCGTGT-3'	5'-AAGATTGTCCAGGCAGAGCC-3'
MyD88	5'-GCATTGAAGAGGACTGCCGA-3'	5'-AGG GGGTCATCTCGAATGGT-3'
TLR-4	5'-GGGTCATGCTTTCTCCGGGT-3'	5'-TTTCACATCTGCACGCAAGGG-3'
18S	5'-CCCACGGAATCGAGAAAGAG-3'	5'-TTGACGGAAGGGCACCA-3'

Table S2: Nucleotide Sequence of the Genes Used in RT-PCR.

Score / Stain	0	1	2	3	4	5
Collagen	Minimal	Very weak	Weak yellow	Moderate	Strong	Very strong

	**yellow** staining	yellow staining	staining	yellow staining	yellow staining	yellow staining
Elastin	Minimal **purple** /black staining	Very weak purple /black staining	Weak purple /black staining	Moderate purple /black staining	Strong purple /black staining	Very strong purple/black staining
Fibrin	Minimal **intense red** staining	Very weak intense red staining	Weak intense red staining	Moderate intense red staining	Strong intense red staining	Very strong intense red staining
Glycans	Minimal **blue** staining	Very weak blue staining	Weak blue staining	Moderate blue staining	Strong blue staining	Very strong blue staining

Table S3: Criteria Used to Score Movat Pentachrome Staining.

<table>
<tr><td rowspan="2">Protein of interest</td><td rowspan="2">Femoral artery/ femoral vein</td><td colspan="2">Experimental group</td><td>Immunopositivity</td><td colspan="2">Immunopositivity (PA vs PV)</td></tr>
<tr><td>Ethanol</td><td>Ethanol + TAK-242</td><td>Ethanol vs ethanol + TAK-242</td><td>Ethanol</td><td>Ethanol + TAK-242</td></tr>
<tr><td>α-SMA</td><td>Artery</td><td>PA>CA</td><td>PA<CA</td><td>More in ethanol group</td><td rowspan="2">PA>PV</td><td rowspan="2">PA<PV</td></tr>
<tr><td></td><td>Vein</td><td>PV<CV</td><td>PV>CV</td><td>More in ethanol + TAK-242</td></tr>
<tr><td>Vimentin</td><td>Artery</td><td>PA>CA</td><td>PA>CA</td><td>Comparable in both group</td><td rowspan="2">PA>PV</td><td rowspan="2">PA~PV</td></tr>
<tr><td></td><td>Vein</td><td>PV<CV</td><td>PV<CV</td><td>Comparable in both group</td></tr>
<tr><td>TGF-β</td><td>Artery</td><td>PA>CA</td><td>PA>CA</td><td>Comparable in both group</td><td rowspan="2">PA<PV</td><td rowspan="2">PA>PV</td></tr>
<tr><td></td><td>Vein</td><td>PV>CV</td><td>PV<CV</td><td>More in ethanol group</td></tr>
</table>

VCAM-1	Artery	PA>CA	PA>CA	More in ethanol + TAK-242	PA<PV	PA~PV
	Vein	PV<CV	PV <CV	More in ethanol + TAK-242		
Cathepsin L	Artery	PA>CA	PA>CA	More in ethanol + TAK-242	PA<PV	PA>PV
	Vein	PV<CV	PV<CV	Comparable in both group		
MMP-9	Artery	PA>CA	PA>CA	More in ethanol group	PA<PV	PA~PV
	Vein	PV>CV	PV~CV	More in ethanol group		
Collagen IV	Artery	PA<CA	PA>CA	More in ethanol + TAK-242	PA<PV	PA<PV
	Vein	PV>CV	PV<CV	More in ethanol + TAK-242		
TLR-4	Artery	PA>CA	PA<CA	More in ethanol group	PA>PV	PA~PV
	Vein	PV<CV	PV<CV	Comparable in both group		
TREM-1	Artery	PA>CA	PA<CA	Comparable in both group	PA<PV	PA<PV
	Vein	PV<CV	PV<CV	Comparable in both group		

MCP-1	Artery	PA>CA	PA>CA	Comparable in both group	PA<PV	PA<PV
	Vein	PV<CV	PV>CV	More in ethanol group		
IL-8	Artery	PA>CA	PA>CA	More in ethanol group	PA>PV	PA~PV
	Vein	PV < CV	PV < CV	More in ethanol + TAK-242		
TNF-α	Artery	PA>CA	PA>CA	More in ethanol + TAK-242	PA<PV	PA~PV
	Vein	PV>CV	PV>CV	Comparable in both group		
IL-6	Artery	PA>CA	PA~CA	More in ethanol group	PA>PV	PA<PV
	Vein	PV < CV	PV ~<CV	More in ethanol + TAK-242		

Table S4: Summary of the expression profile of the protein of interest in the femoral artery and femoral vein on AVF and contralateral side. PA- femoral artery proximal to the site of anastomosis, PV- femoral vein proximal to the site of anastomosis, CA- contralateral femoral artery, CV- contralateral femoral vein, ~comparable or approximately equal, < less than, > more than. AVF: Arteriovenous fistula, IL: interleukin, MCP-1: monocyte chemotactic protein 1, MMP: matrix metalloproteinases, α-SMA: alpha-smooth muscle actin, TGF- β: transforming growth factor-beta, TNF-α: tumor necrosis factor-alpha, TREM-1: triggering receptor expressed on myeloid cells-1, TLR-4: toll-like receptor-4, VCAM-1: vascular cell adhesion molecule 1.

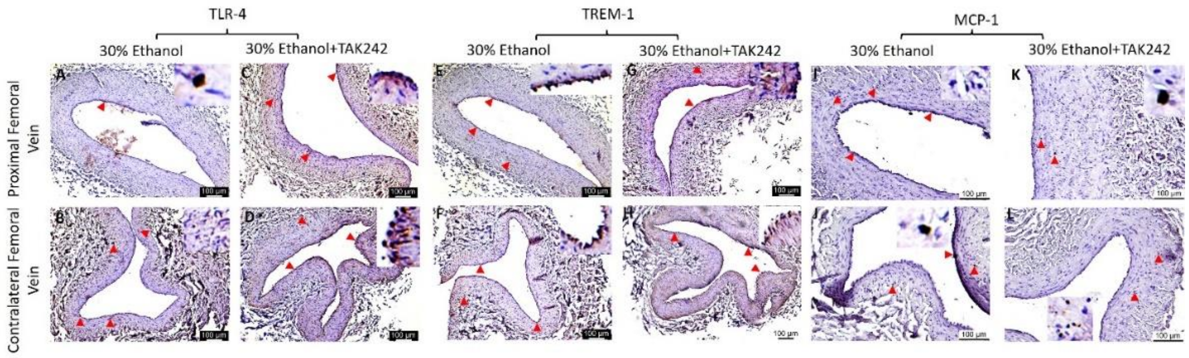

Figure S1: Immunohistochemistry for toll-like receptor (TLR)-4, triggering receptor expressed on myeloid cells (TREM)-1, and monocyte chemoattractant protein (MCP)-1 in the femoral vein in ethanol and ethanol + TAK-242 group. TLR-4 staining (panels A to D), TREM-1 staining (panel E to H), and MCP-1 staining (panels I to L) in the proximal femoral vein and contralateral femoral vein. The red arrowheads show positively stained cells and inserts show images in higher magnification. There are representative images from all animals in the study.

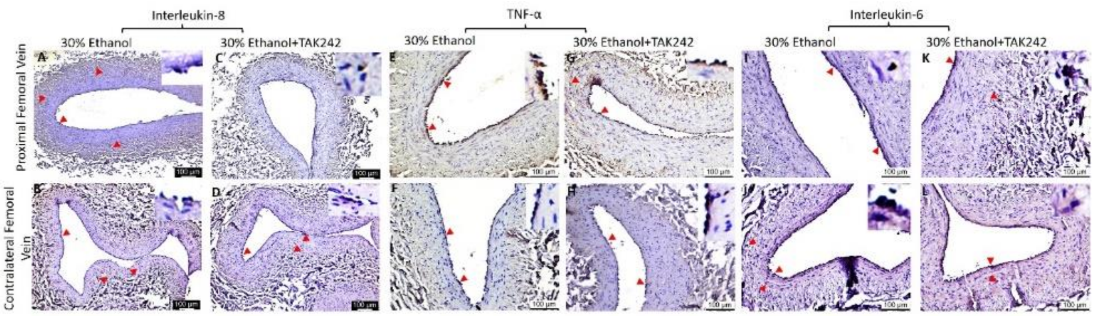

Figure S2: Immunohistochemistry for interleukin (IL)-8, tumor necrosis factor (TNF)-α, and IL-6 in the femoral vein in ethanol and ethanol + TAK-242 group. IL-8 staining (panels A to D), TNF-α staining (panel E to H), and IL-6 staining (panels I to L) in the proximal femoral vein and contralateral femoral vein. The red arrowheads show positively stained cells and inserts show images in higher magnification. There are representative images from all animals in the study.

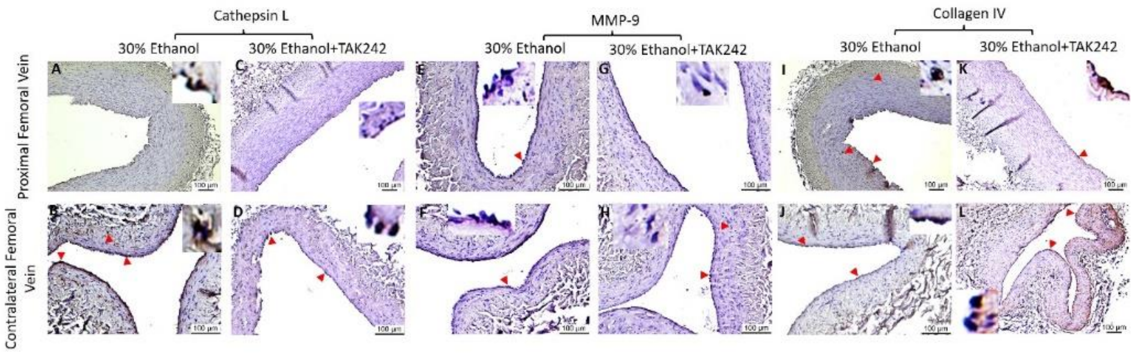

Figure S3: Immunohistochemistry for cathepsin L, matrix metalloproteinases (MMP)-9, and collagen IV in the

femoral vein in ethanol and ethanol + TAK-242 group. Cathepsin L staining (panels A to D), MMP-9 staining (panel E to H), and collagen IV staining (panels I to L) in the proximal femoral vein and contralateral femoral vein. The red arrowheads show positively stained cells and inserts show images in higher magnification. There are representative images from all animals in the study.

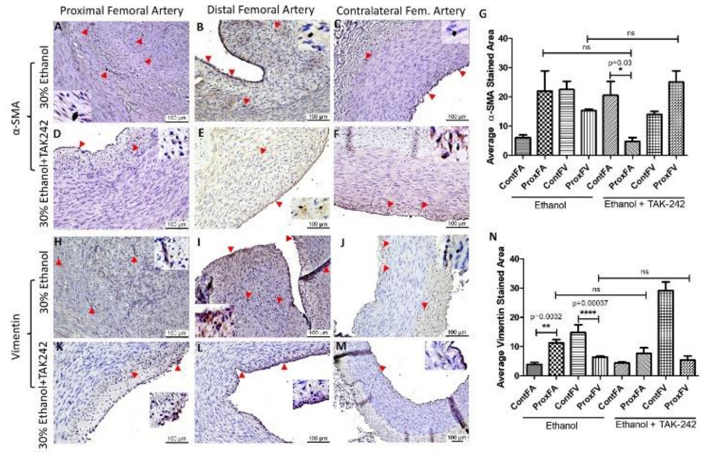

Figure S4: Immunohistochemistry for alpha-smooth muscle action (α-SMA) and vimentin in the femoral artery in ethanol and ethanol + TAK-242 group. α-SMA staining (panels A to F) and vimentin staining (panel H to M) in the proximal femoral artery, distal femoral artery, and contralateral femoral artery. Average stained intensity for α-SMA (panel G) and vimentin (panel N). The red arrowheads show positively stained cells and inserts show images in higher magnification. There are representative images from all animals in the study. Data are presented as mean ± SEM (n=3 in ethanol and n=4 in ethanol + TAK-242). *p<0.05, **p<0.01 ****p<0.0001.

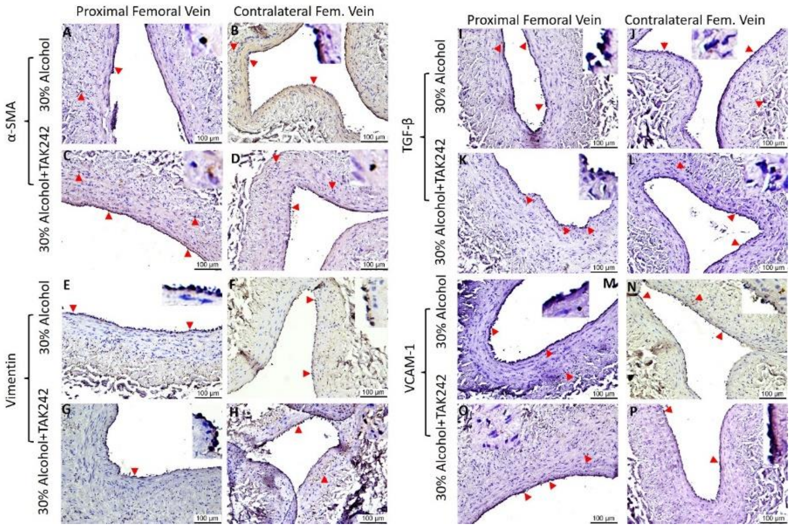

Figure S5: Immunohistochemistry for alpha-smooth muscle action (α-SMA), vimentin, transforming growth factor-beta (TGF-β), and vascular cell adhesion molecule (VCAM)-1 in the femoral vein in ethanol and ethanol + TAK-242 group. α-SMA staining (panels A to D) and vimentin staining (panels E to H), TGF-β staining (panels I to L), and VCAM-1 staining (panels M to P) in the proximal femoral vein and contralateral femoral vein. The red

arrowheads show positively stained cells and inserts show images in higher magnification. There are representative images from all animals in the study.

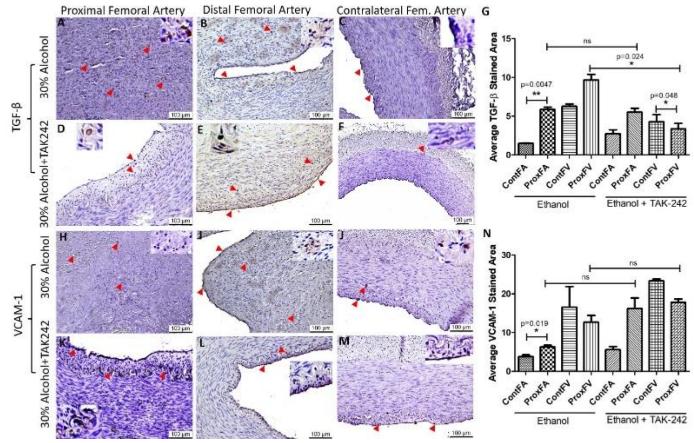

Figure S6: Immunohistochemistry for transforming growth factor-beta (TGF-β) and vascular cell adhesion molecule (VCAM)-1 in the femoral artery in ethanol and ethanol + TAK-242 group. TGF-β staining (panels A to F) and VCAM-1 staining (panel H to M) in the proximal femoral artery, distal femoral artery, and contralateral femoral artery. Average stained intensity for TGF-β (panel G) and VCAM-1 (panel N). The red arrowheads show positively stained cells and inserts show images in higher magnification. There are representative images from all animals in the study. Data are presented as mean ± SEM (n=3 in ethanol and n=4 in ethanol + TAK-242).*p<0.05, **p<0.01.

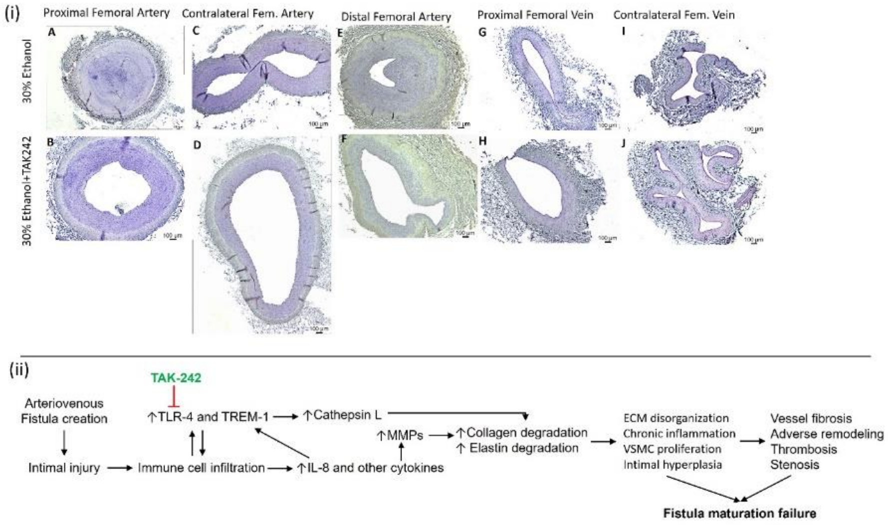

Figure S7: (i) Full area sections of the proximal femoral artery (panels A and B), distal femoral artery (panels E and F), proximal femoral vein (panels G and H), contralateral femoral artery (panels C and D), and vein (panels I and J) in ethanol and ethanol + TAK-242 group. There are representative images from all animals in the study. (ii) Flow schematics of the study hypothesis.
